# Synthesis and characterization of Na-P_1_ (GIS) zeolite using a kaolinitic rock

**DOI:** 10.1038/s41598-021-84383-7

**Published:** 2021-03-01

**Authors:** Daniela Novembre, Domingo Gimeno, Alessandro Del Vecchio

**Affiliations:** 1 Dipartimento di Ingegneria e Geologia, Università degli Studi “G.D’Annunzio”, Via dei Vestini 30, 66013 Chieti, Italy; 2 Departamento de Mineralogia, Petrologia i Geologia Aplicada, Universitat de Barcelona, 08028 Barcelona, Spain

**Keywords:** Mineralogy, Characterization and analytical techniques

## Abstract

This work focuses on the hydrothermal synthesis of Na-P_1_ zeolite by using a kaolinite rock coming from Romana (Sassari, Italy). The kaolin is calcined at a temperature of 650 °C and then mixed with calculated quantities of NaOH. The synthesis runs are carried out at ambient pressure and at variable temperatures of 65 and 100 °C. For the first time compared to the past, the Na-P1 zeolite is synthesized without the use of additives and through a protocol that reduces both temperatures and synthesis times. The synthesis products are analysed by X-ray diffraction, high temperature X-ray diffraction, infrared spectroscopy, scanning electron microscopy and inductively coupled plasma optical emission spectrometry. The cell parameters are calculated using the Rietveld method. Density and specific surface area are also calculated. The absence of amorphous phases and impurities in synthetic powders is verified through quantitative phase analysis using the combined Rietveld and reference intensity ratio methods. The results make the experimental protocol very promising for an industrial transfer.

## Introduction

Zeolites are a group of tectosilicates of about 50 minerals with synthetic analogues. Their structure is made of three-dimensional networks of Al/Si tetrahedra arranged to form channels containing water and exchangeable alkaline or alkaline earth cations. Thanks to these peculiar structural characteristics, they are widely used in separation and refinery industries as catalysts, adsorbents and ion exchangers.

The zeolite P class has the typical oxide formula: M_2/n_O^.^Al_2_O_3_^.^1.80–5.00SiO_2_.5H_2_O where M is a n-valent cation, normally an alkali metal^1^. P zeolites are characterised by water-softening properties over zeolite A^2^ and are used for the formation of environmental- friendly detergents^3^. Na-P is also useful for gas separation applications^4, 5^ and for removal of toxic and radioactive waste species^1, 6–8^. Synthetic zeolite Na-P_1_ is also recognized as a better sorbent of Cr (VI) than Clinoptilolite^9^.

Despite having variable Si/Al ratios, all P zeolites are characterised by the same framework topology, which is the GIS net, gismondine, according to Meier and Olson^10^. Three polymorphs are recognized for Na-P: the cubic phase called Na-P_1_, refined in *I*_*4*_^11^ and with a unit cell content of Na_6_Al_6_Si_10_O_32_^.^12H_2_O; the orthorhombic one, the so-called Na-P_2_ with formula Na_4_Al_4_Si_12_O_32_^.^14H_2_O, crystallizing in *Pnma*^12^; the tetragonal one for high silica variety of Na-P (Na_3.6_Al_3.6_Si_12.4_O_32_^.^14H_2_O), refined in *I4*_*1*_*/amd*^5^. Another zeolite P with a strict Si/Al ratio of 1.0 is called aluminum P (MAP), whose structure was firstly refined by Albert et al.^13^ in the monoclinic system, space group *C2/c* (Na_8_Al_8_Si_8_O_32_^.^ 15.2H_2_O). Nery et al.^1^ refined the structure of low-silica Cd-MAP, Mn-MAP, Ba-MAP, Sr-MAP and Pb-MAP.

Zeolite P can be synthesized from various methods such as the hydrothermal method^1, 8, 12–16^, the microwave technique^17^, the sol–gel process^18^ and the sonochemical method^19^. In order to reduce the costs of synthesis protocols, alternative synthesis precursors have been tested in place of expensive chemical reagents used in the past, like fly ash^20–22^, kaolinite^15, 23^, clays^18, 24^, rice husk silica^25^ and nuclear wastes^26^.

The scope of the present work is to test a natural rock, *i. e.* a kaolinitic rock coming from Romana (Italy) in the synthesis of monomineralic powders of Na-P_1_. Among clay minerals, in fact, kaolinite is the most common phyllosilicate involved in successful zeolitic synthesis because of its particularly ample/large supply and availability and the well-known reactivity of thermally treated kaolin clays (metakaolin) with alkali^3, 27–31^.

Lovat et al.^23^ just investigated the reaction of metakaolinite (calcination temperature 800 °C) in presence of fluoride ions at 85 °C founding peaks of zeolite P after long periods (60 days reaction time). More recently, Li et al.^15^ synthesized Na-P in 48 h at 180 °C starting from metakaolin (calcinated at 800 °C) by using NaF as structure-directing agent.

In this paper we present the results of a research carried out to define the most favorable conditions for the Na-P_1_ synthesis from metakaolin. The aim is to improve previous mineral synthesis attempts starting from kaolinite; i.e. by developing a synthesis protocol that does not include the use of a structure-directing agents like NaF^15^, and secondly working on the reduction of both the calcination and synthesis temperatures and the synthesis times. An aspect that we also want to deepen in this work is the definition of the degree of purity of the synthesized powders expressed in terms of absence of amorphous phase and impurities coming from the natural kaolinite sample. In the past, Li et al.^15^ defined the synthesized zeolite as “pure” by utilizing the main X-ray diffraction peak of the mineral, i.e., through the use of a formula that calculates the degree of crystallinity of a powder as the ratio between the peak area of product divided by the peak area of referent sample. On the other hand, we believe systematic samplings during the experimental run enable the progress in the crystallization of the mineral to be traced/followed and allows to determine the time at which the climax in the crystallization is reached due to the absence of other phases or amorphous phases. The degree of purity of the synthesized zeolite is here defined through a quantitative phase analysis approach using the combined Rietveld and reference intensity ratio methods.

A natural, cost effective starting material makes this route especially attractive when expanded to an industrial scale as long as the material properties of the synthesized products remain satisfactory, in terms of chemical composition and purity of powders.

## Materials and methods

The kaolin used in this study was collected in a mine located in Romana (Sassari province, Italy). For the chemical composition of kaolin and its mineralogical, morphological and spectroscopic characterization, see Novembre et al.^30^. The kaolin was triturated, and the sandy fraction was separated by retention in a sieve. The fraction below 90 μm was then collected, suspended in distilled water, sonicated, and centrifuged for separation of the silt fraction and collection of the clay fraction. Preliminary calcination of kaolin was carried out using the following procedure: aliquots of kaolin were placed in open porcelain crucibles which were heated in a Gefran Model 1200 furnace (Gefran Spa, Brescia, Italy) to the calcination temperature (650 °C) at a pressure of 1 atm. The heating rate of the sample was 1.5 °C s^−1^. Once the calcination temperature was reached, the crucibles were left in the furnace for 2 h and then removed and cooled at room temperature. The NaOH used in the synthesis protocol were purchased from Riedel-de Haën (Honeywell Riedel-de Haën, Bucharest, Romania). The purity of the reagent was of 99%. 2 g of metakaolinite have been dissolved in 20 ml of a NaOH (8%) solution. The initial mixture had the composition: 6.25 SiO_2_–1.00 Al_2_O_3_–3.6 Na_2_O. The mixture was homogenized for two hours with a magnetic stirrer. Then was put inside a stainless-steel hydrothermal reactor and heated at 10 °C/min until the desidered temperature (65 and 100 °C) and kept for different times. Synthesis products were sampled periodically from the reactor, filtered with distilled water and dried in an oven at 40 °C for a day.

Metakaolinite and products of synthesis were analysed by powder X-ray diffraction (XRPD); the instrument was a Siemens D5000 operating with a Bragg–Brentano geometry (CuKα = 1.518 Å, 40 kV, 40 mA, 2°–45°, 2°–90° 2theta scanning interval, step size 0.020° 2theta). Metakaolinite revealed an amorphous character by the XRD analysis. Identification of Na-P and relative peak assignment was performed with reference to the following JCPDS code: 00-039-0219. Both the crystalline and amorphous phases in the synthesis powders were estimated using quantitative phase analysis (QPA) applying the combined Rietveld and reference intensity ratio (RIR) methods; corundum NIST 676a was added to each sample, amounting to 10% (according to the strategy proposed by Novembre et al.^32^ and the powder mixtures were homogenized by hand-grinding in an agate mortar. Data for the QPA refinement were collected in the angular range 5–120° 2theta with steps of 0.02° and 10 s step^−1^, a divergence slit of 0.5° and a receiving slit of 0.1 mm.

Data were processed with the GSAS software^33^ and the graphical interface^34^ starting with the structural models proposed by Albert et al.^13^ for Na-P_1_ and Gatta et al. for nepheline^35^. The following parameters were refined: background parameters, zero shift, cell parameters and peak profiles.

Thermal stability and phase transformations were studied using high-temperature X-ray diffractometry with a PANalytical X’Pert PRO MPD (CuKα = 1.518 Å, 45 kV, 40 mA, X’Celerator Detector with active length of 2.122°, θ/2θ scan from 5° to 50° 2θ with step size of 0.017° and measuring time of 100 s per step), equipped with a high temperature camera Anton Paar HTK1200N (thermocouple Pt 10% RhPt). The sample holder was a platform with a 16 mm diameter equipped with a ceramic cup (0.8 mm deep and 14 mm inner diameter) for holding powder. The analyses were taken at different temperatures: from 28 °C up to 1000 °C, every 100 °C. Slope was 10 °C/min. The program package GSAS—EXPGUI was used for the calculation of cell parameters, using the Rietveld full-profile method starting with the structural models proposed by Albert et al.^13^ for Na-P_1_ and Gatta et al.^35^ for nepheline.

Morphological analyses were obtained by means of scanning electron microscopy (JEOL JSM-840 served by a LINK Microanalysis EDS system, with operating conditions of 15 kV and window conditions ranging from 18 to 22 mm)^36^.

Induced coupled plasma optical emission spectroscopy technique (ICP-OES, Perkin Elmer Optima 3200 RL) was conducted on synthesized powders through previous fusion (Pt meltpot) in lithium meta-tetra borate pearls and subsequent acid solubilisation and analytical determination^37^.

Zeolite density was calculated by He-picnometry using an AccuPyc 1330 pycnometer. The specific surface and porosity were obtained by applying the BET (Brunauer–Emmett–Teller) method with N_2_ using a Micromeritics ASAP2010 instrument (operating from 10 to 127 kPa)^38^.

The infrared analysis was performed with a spectrometer FTLA2000, served by a separator of KBr and a DTGS detector; the source of IR radiation was a SiC (Globar) filament. Samples were treated according the method of Novembre et al.^39, 40^ using powder pressed pellets (KBr/sample ratio of 1/100, pressure undergone prior determination 15 t/cm^2^); spectra were processed with the program GRAMS-Al (GRAMS/AI Spectroscopy Software, Thermo Scientific Company).

Differential thermal analysis (DTA) and thermogravimetry (TG) were performed on the zeolitic powder using a Mettler TGA/SDTA851e instrument (10°/min, 30–1100 °C, sample mass of ~ 10 mg, Al_2_O_3_ crucible) (Mettler Toledo, Greifensee, Switzerland)^41^.

## Results

**Figure 1:**
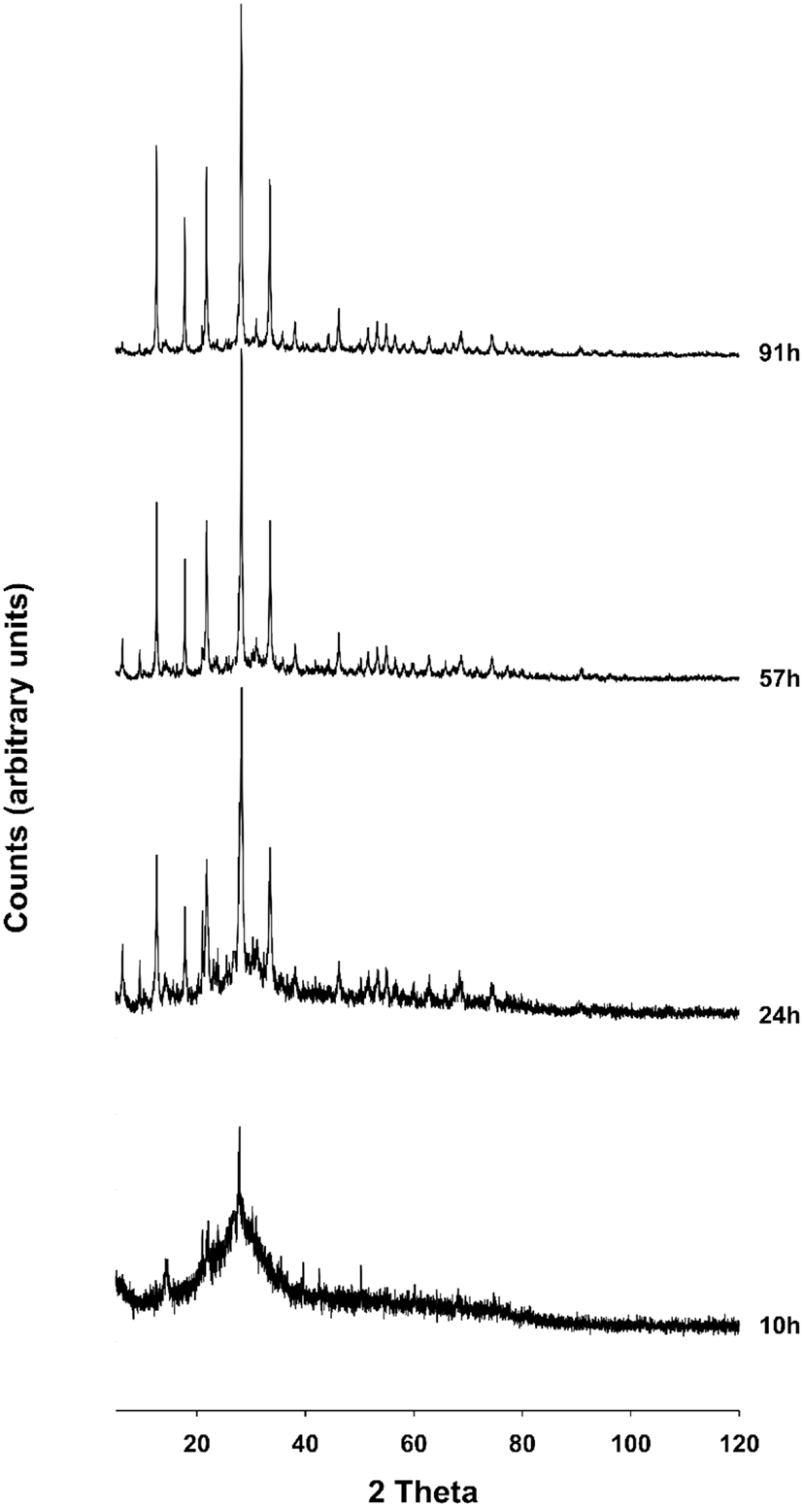
X-ray diffractometric sequence of the synthesis run at 65 °C.

**Figure 2:**
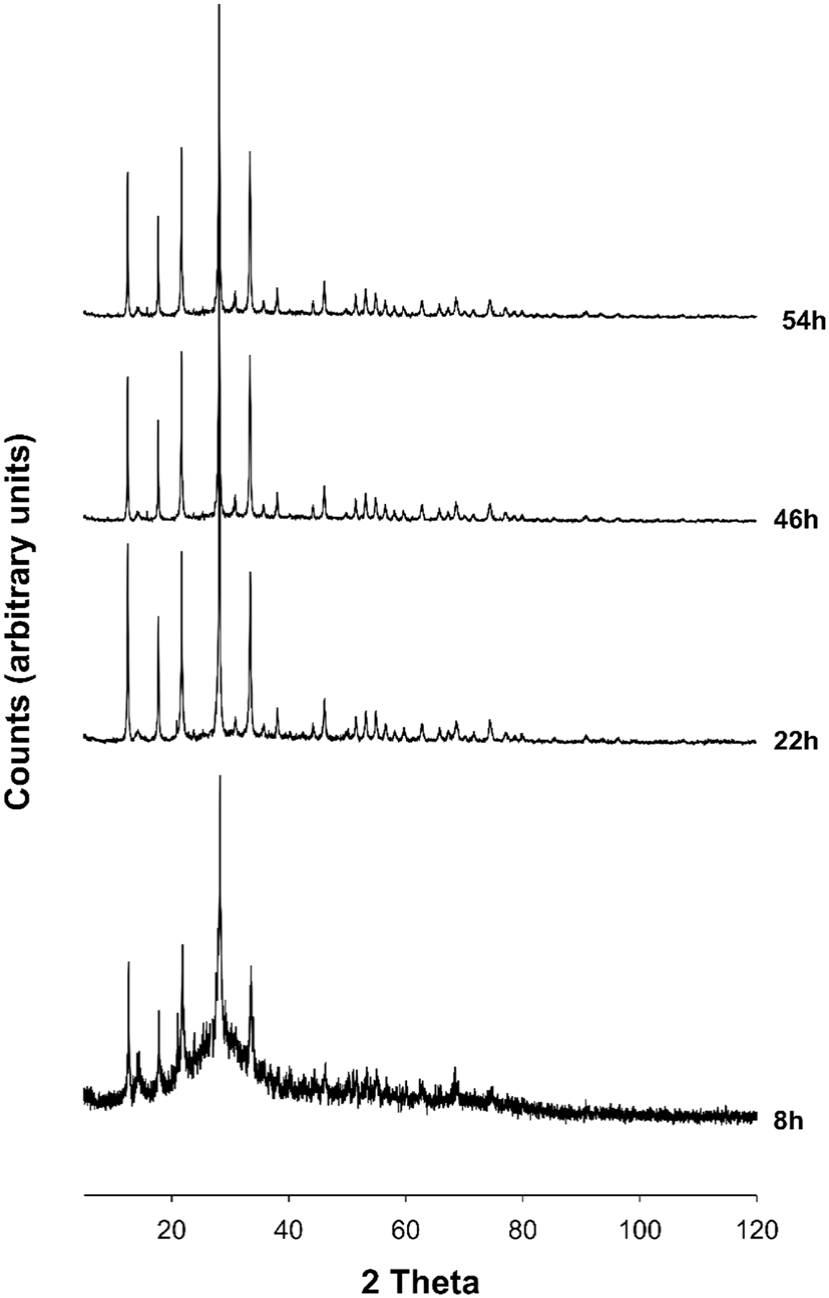
X-ray diffractometric sequence of the synthesis run at 100 °C.

Results of XRPD analyses performed on the two synthesis runs conducted at 65 °C and 100 °C are illustrated in Figs.1, 2, respectively.

**Figure 3:**
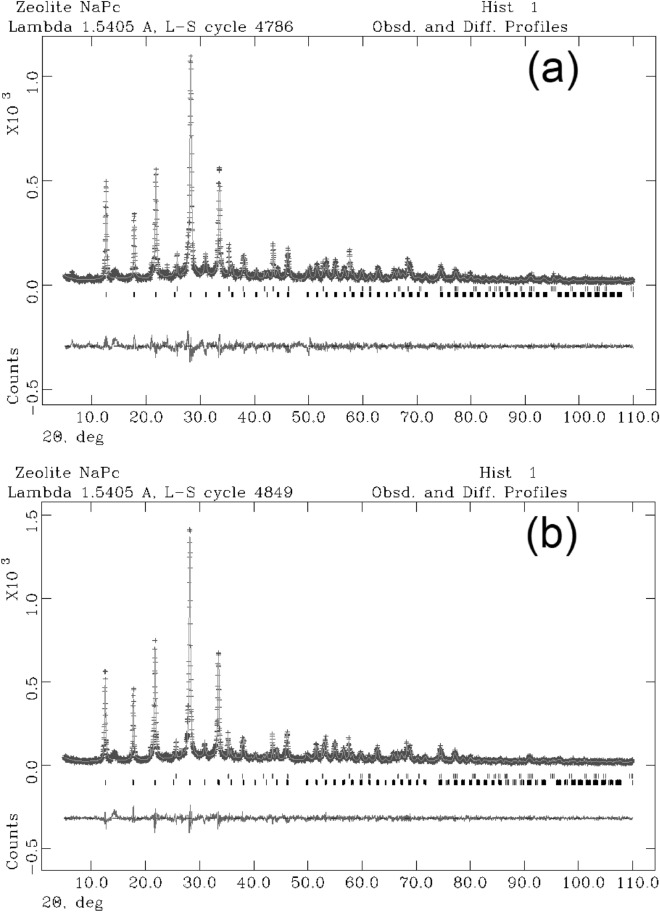
Rietveld refinement plot: observed (+) and calculated profiles and difference plot for Na-P_1_ zeolite and corundum NIST 676a with tick marks at the position of the Bragg peaks. From the bottom: Na-P_1_ zeolite, curundum NIST 676a. (**a**) Sample at 65 °C; (**b**) Sample at 100 °C.

Analyzing the two synthesis runs carried out at 65 and 100 °C, it is clear that the best results are obtained in the second case. In fact, in the synthesis conducted at 65 °C, the presence in the XRD spectrum of small reflections below 10° of 2 theta is observed, probably referable to intermediate metastable phases; in fact, these peaks tend to decrease considerably in intensity passing to 91 h, and this leads us to think that with the prolongation of the heat treatment these phases are eliminated. The synthesis run carried out at 100 °C, on the other hand, shows the absence of such peaks and leads us to think that the effect of raising the synthesis temperature from 65 to 100 °C is unfavorable to the formation of these phases. When analyzing the experiment performed at 65 °C, appearance of Na-P_1_ zeolite is evident at 24 h. The existence field of the NaP_1_ zeolite is very large; peaks grow in height until reaching the maximum intensity at 91 h. Results of the QPA analyses conducted on samples at 24, 57 and 91 h are illustrated in Table 1. The zeolitic percentage increases over time to the detriment of the amorphous component and reaches its climax at 91 h (91.98%). For the sample at 91 h the observed and calculated profiles and difference plots for Na-P_1_ and corundum NIST 676a are reported in Fig. 3a.

**Table 1:** Results of the QPA analyses conducted on samples synthesized at 65° and 100 °C.

Temperature	65 °C			100 °C			
sample + 10% corundum Nist 676a	24 h	57 h	91 h	8 h	22 h	46 h	54 h
R_wp_	0.17	0.19	0.2	0.16	0.17	0.16	0.16
R_p_	0.13	0.14	0.15	0.12	0.13	0.12	0.12
CHI^2^	1.76	2.1	2.14	1.52	1.65	1.65	2.39
Space group NaP	*C2/c*	*C2/c*	*C2/c*	*C2/c*	*C2/c*	*C2/c*	*C2/c*
*a* (Å)	14.23311 (0.0051)	14.1933 (0.0039)	14.2028 (0.0045)	14.1963 (0.0057)	14.1892 (0.0037)	14.1553 (0.0049)	14.1983 (0.0049)
*b* (Å)	10.0455 (0.0005)	10.0572 (0.0006)	10.0539 (0.0005)	10.0525 (0.0005)	10.0593 (0.0007)	10.0544 (0.0004)	10.0344 (0.0004)
*c* (Å)	10.0528 (0.0007)	10.0372 (0.0006)	10.0394 (0.0009)	10.0262 (0.0008)	10.0291 (0.0008)	10.0283 (0.0009)	10.0833 (0.0009)
% amorphous	85.37 (11)	40.85 (12)	8.02 (13)	93.86 (8)	45.92 (19)	27.49 (16)	7.3 (16)
NaP	12.86 (14)	65.95 (19)	91.98 (14)	6.14 (17)	54.08 (18)	72.51 (18)	92.7 (18)

The crystallization of Na-P_1_ zeolite is verified by the PXRD analyses (Fig. 2) in the time interval 8–54 h in the experiment performed at 100 °C. About 50% of the crystallization of the Na-P_1_ zeolite takes place in the first 22 h of the synthesis run (Table 1). The climax in the crystallization of the zeolite is reached at 54 h with the achievement of 92.7% of zeolitic phase. The observed and calculated profiles and difference plots for Na-P_1_ and corundum NIST 676a have been performed for the sample at 54 h (Fig. 3b).

Cell parameters of Na-P_1_, refined with orthorombic simmetry space group *Pna2*_*1*_, remain constant within error as a function of the experimental run time both at 65 and 100 °C (Table 1). The results of the Rietveld refinements provide cell values that are in good agreement with the structural model proposed by Albert et al.^13^.

**Figure 4:**
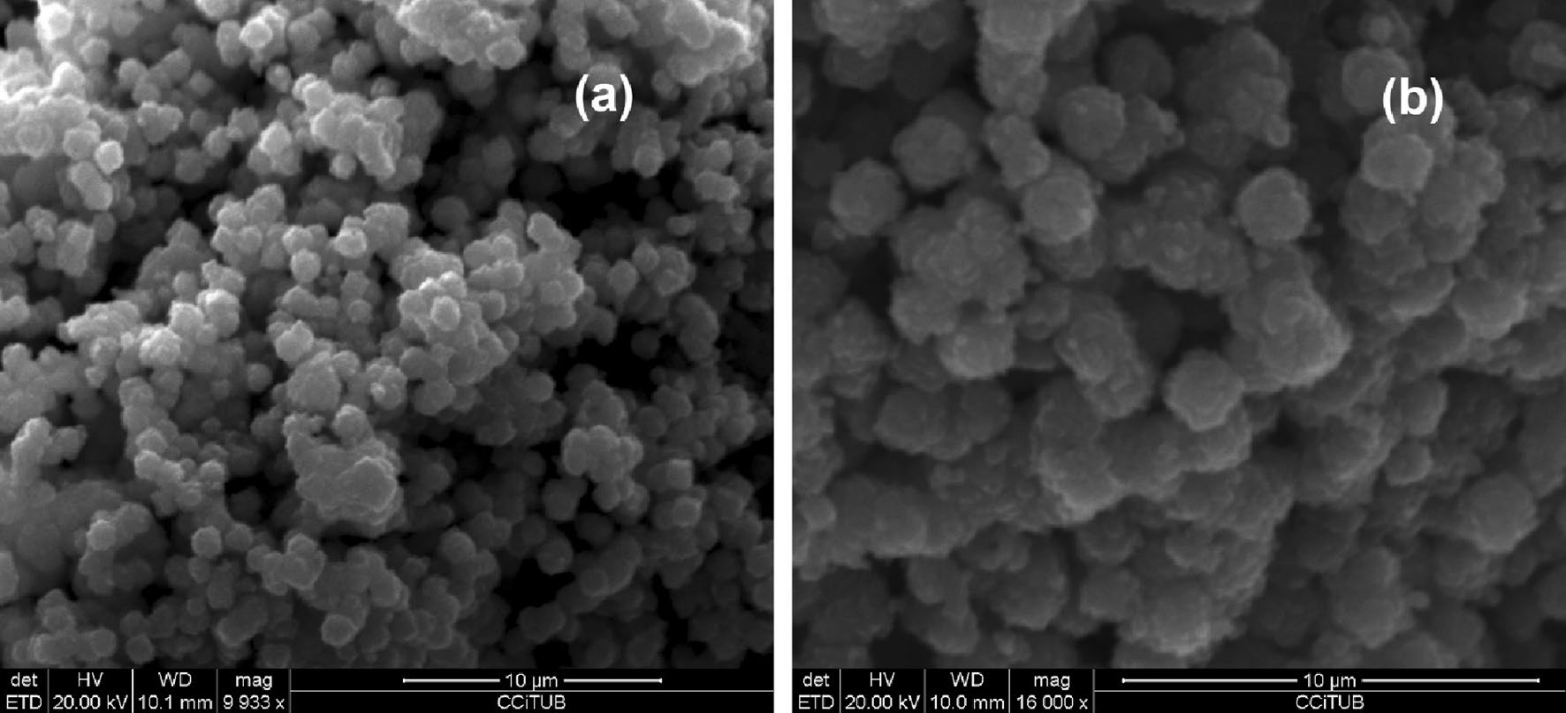
SEM image of Na-P_1_ zeolite crystals obtained at 91 h (65 °C) (**a**) and at 54 h (100 °C) (**b**) of the synthesis runs.

Figure 4a,b reports SEM images of Na-P_1_ crystals from experimental runs performed at 65° (91 h) and 100 °C (54 h), respectively. It results a ball-like morphology of the crystals and an average maximum length of crystals around 1–2 µm.

Chemical analysis performed on samples at 91 h (65 °C) and 54 h (100 °C) resulted in the stoichiometry of Na_6.00_Al_5.94_Si_10.02_O_32_ and Na_6.02_Al_5.91_Si_10.03_O_32_ respectively. The density of Na-P_1_ from samples at 91 h (65 °C) and 54 h (100 °C) was determined to be 2.031(5) and 2.042 (4) g/cm^3^, respectively, in good agreement with Breck^42^.

**Figure 5:**
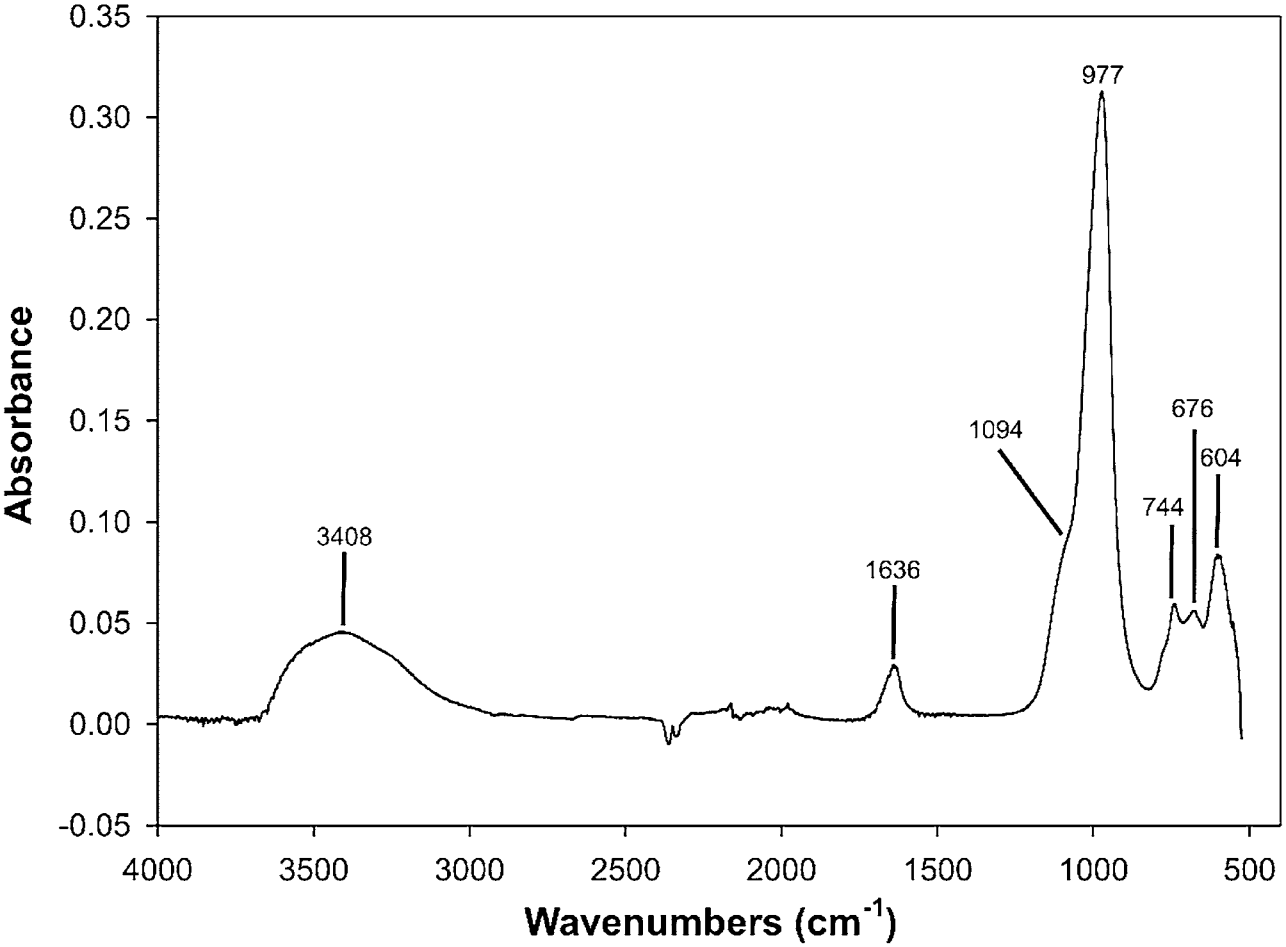
IR spectrum of the Na-P_1_ zeolite at 91 h (100 °C).

Further characterizations were carried out on the sample at 91 h (65 °C). Figure 5 illustrates the infrared spectrum of the sample. The significant broad peaks are located at 3408 and 1636 cm^−1^ for O–H stretching and bending, respectively. The band at 1094 is assigned to to the asymmetric stretching vibration due to external linkages between tetrahedra, structure sensitive, sensu Flaningen et al.^43^ and that at 977 cm^−1^ is assigned to the asymmetric stretching vibration caused by internal vibrations of the framework SiO_4_. The bands at 744 and 676 cm^−1^ are attributed to Si–O–Si symmetric stretching vibration of internal tetrahedron. The peak at 604 cm^−1^ is attributed to the double rings vibration. Data are coherent with those available in the literature^8, 13, 43^.

**Figure 6:**
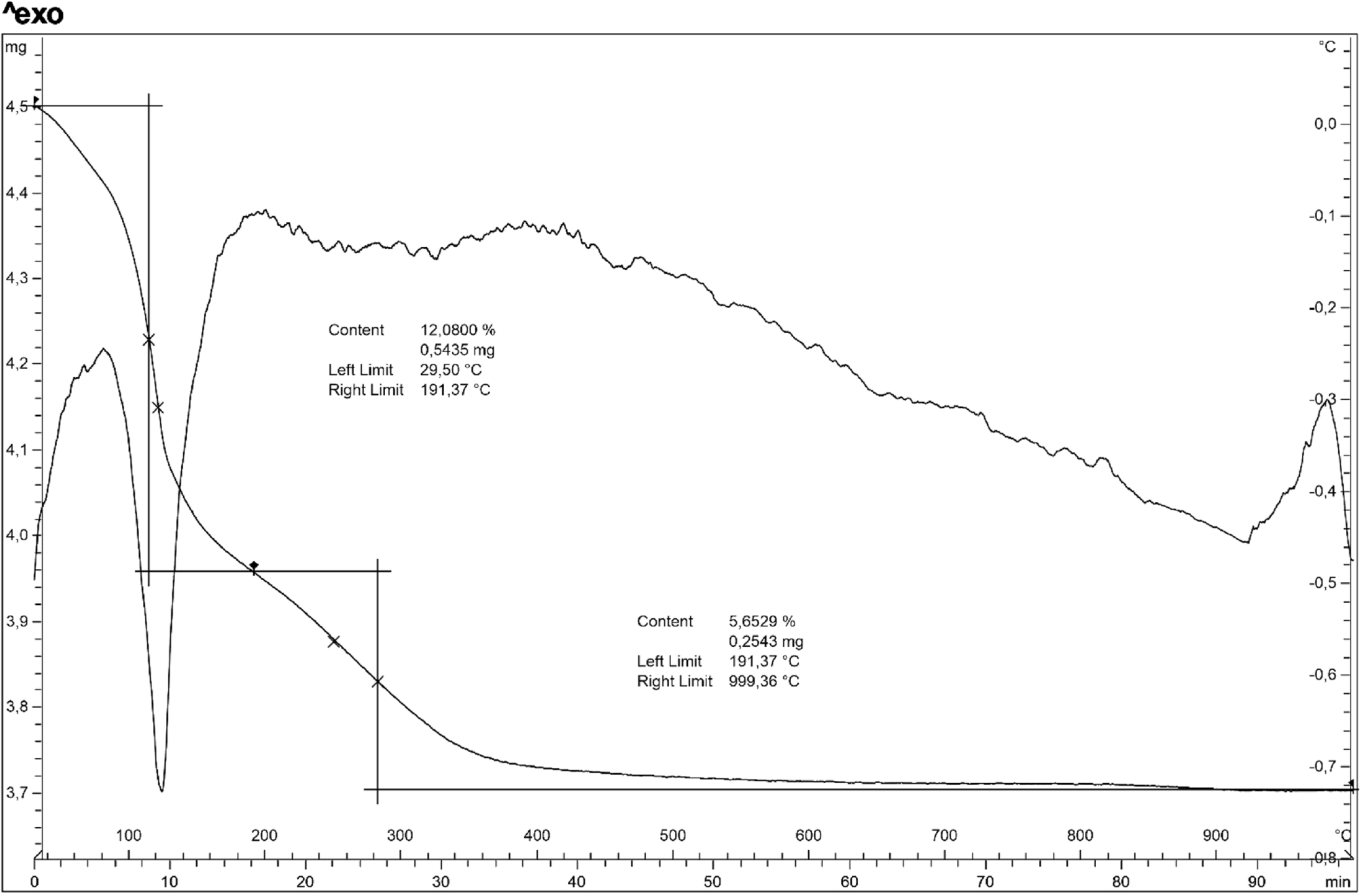
DTA-TG analysis of the sample at 91 h (100 °C).

Thermogravimetric analysis conducted on sample at 91 h (65 °C) revealed a gradual and continuous water loss up to 1000 °C (Fig. 6). In particular it indicates a two-stage mass loss. At the first stage, c.a. 12% loss occurs at about 191 °C; this was reasonable due to the loss of the adsorbed water. The second occurred between 191 and 850 °C with a mass loss of 5.6% and is related to the removal of the crystal water. The endothermic peaks revealed by the DTA curve at 122 °C reflects the dehydration process and is in agreement with findings by Zubowa et al. and Huo et al.^8, 17^. At about 970 °C there is an exothermic peak which testifies the transformation into a new phase.

**Figure 7:**
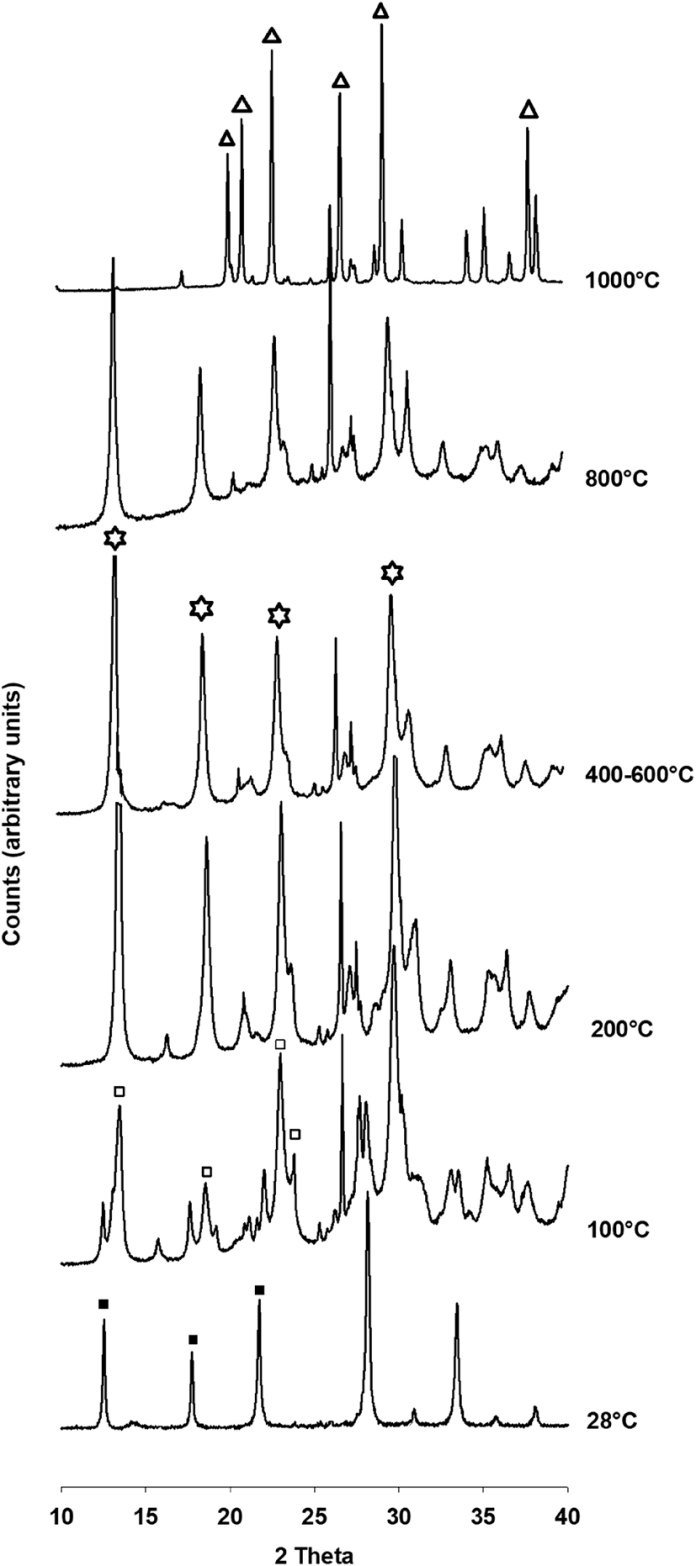
High temperature XRD pattern of sample at 91 h (100 °C). Full squares: Na-P_1_; empty squares: tetragonal NaP; empty stars : phillipsite; full stars: nepheline.

The high temperature X ray diffraction on sample at 91 h (65 °C) (Fig. 7) reveals the lower of simmetry to tetragonal NaP structure, just visible at 100 °C; then the tetragonal phase transforms into phillipsite in the temperature interval 400–800 °C, and finally the passage to the stable phase of nepheline is testified at 1000 °C. The phase transitions observed are in agreement with the findings of Huo et al.^8^. The exothermic peak revealed by the DTA curve is indicative of the crystallisation of nepheline. Rietvield refinement of sample at 1000 °C provides crystallographic values of a = b = 10.15 Å, c = 8,44 Å, α = β = 90°, γ = 120°.

**Figure 8:**
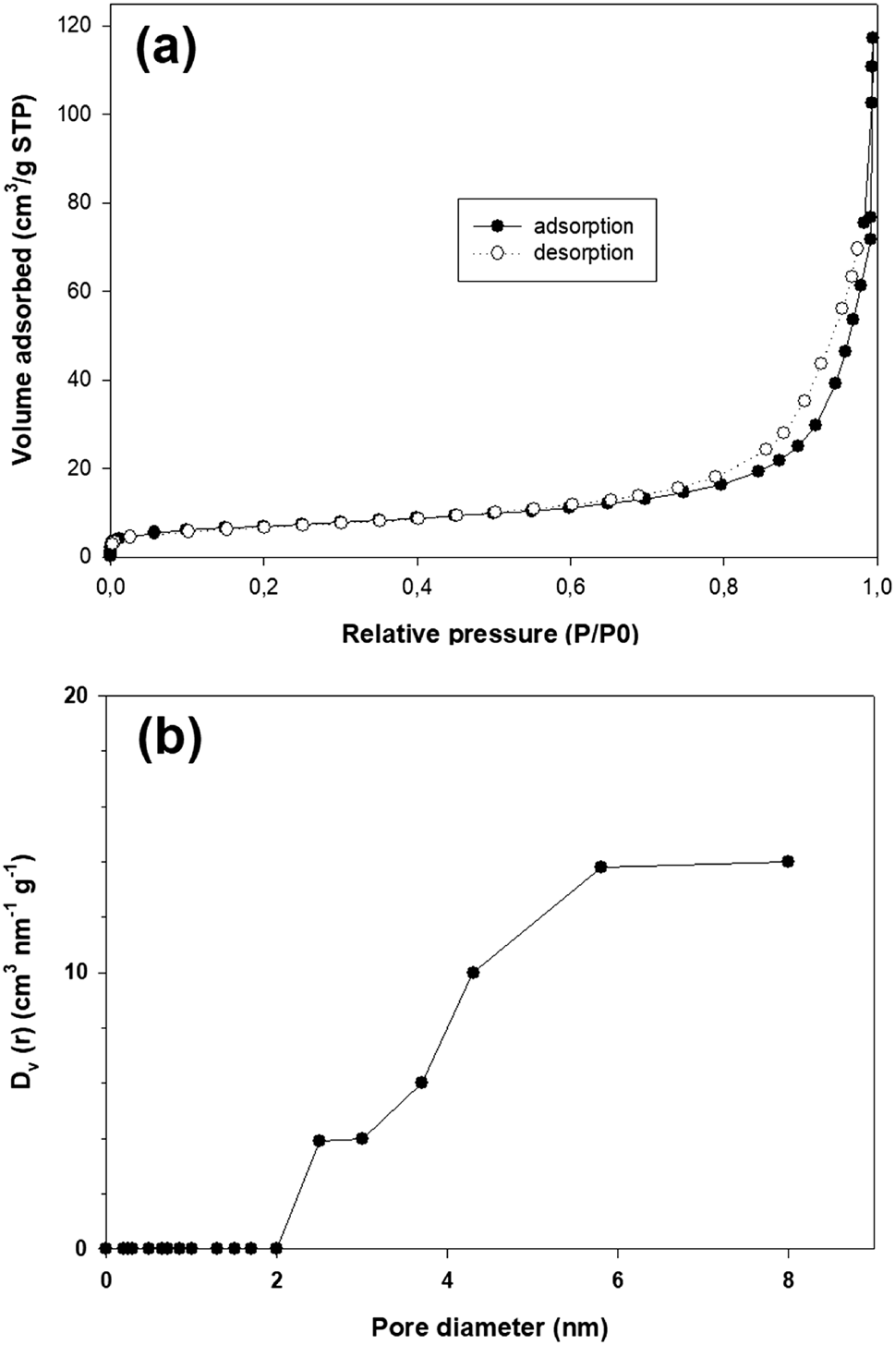
(**a**) Nitrogen adsorption–desorption isotherms of the Na-P_1_ zeolite and (**b**) pore size distribution pattern.

Figure 8a shows the N_2_ adsorption–desorption plots at 77 K for zeolite Na-P_1_. There is evidence of a hysteresis loop indicating the presence of mesopores; the vertical hysteresis loop indicates cylindrical mesopores. There is a steep increase in the adsorbed amount of nitrogen when the relative pressure (P/P0) is higher than 0.8. Micropore-size distribution was calculated according to the method proposed by Horvath and Kawazoe (Fig. 8b). The absence of an inflection point demonstrates the absence of micropores in the sample analyzed.

## Conclusion

This work describes the synthesis of zeolite Na-P_1_ using a kaolinitic rock. Appearance of Na-P_1_ phase begins at about 24 h at 65 °C and at about 8 h at 100 °C. The existence field of the Li-A(BW) zeolite is very large, in fact no phase replaces the zeolite in the time interval 1–91 h at 65 °C and 1–54 h at 100 °C. The chemical-physical, morphological and spectroscopic characterization of experimental products proved the efficacy of the experimental procedure proposed here.

When our results are compared with those of other authors who have synthesized the same zeolite starting from a natural precursor, a reduction of the calcination temperature of kaolinite, of synthesis temperature, and crystallization time is evident. Lovat et al.^23^ and Li et al.^15^ in fact, operate a calcination temperature of kaolinite of 800 °C, while we reduced it to 650 °C. In addition, the same authors investigated the reaction of metakaolinite in presence of fluoride ions; here the synthesis protocol does not include the use of these additives. Another important observation is the reduction of synthesis times, which in our case makes the experimental synthesis protocol very attractive from an economic, and therefore industrial, point of view. In fact, the previous authors synthesize the Na-P_1_ zeolite at 85 °C in 60 days^23^ and at 180 °C in 48 h^15^. In the present research the zeolite instead appears at 65 °C already at 24 h and at 100 °C at 8 h. Another substantial difference between our work and that of previous authors lies in the effective assessment of the degree of success of the experiment from calculation by QPA of the percentage of crystallization vs. amorphous material and other impurities. The industry requires at least 90% pure products. And our powders reach 91.98% purity at 65 °C (91 h) and 92.7% at 100 °C (54 h). The results of the QPA analyses and the wide temporal range of stability of this zeolite suggests that transfer to an industrial production scale would be possible.
